# MOSAIC CHROMOSOMAL ALTERATIONS AND LONGEVITY

**DOI:** 10.1093/geroni/igac059.523

**Published:** 2022-12-20

**Authors:** Anastasia Leshchyk, Qingyan Xiang, Stefano Monti, Thomas Perls, Stacy Andersen, Anastasia Gurinovich, Zeyuan Song, Paola Sebastiani

**Affiliations:** Boston University, Boston, Massachusetts, United States; Boston University, Boston, Massachusetts, United States; Boston University, Boston, Massachusetts, United States; Boston University, Boston, Massachusetts, United States; Boston University School of Medicine, Boston, Massachusetts, United States; Tufts Medical Center, Boston, Massachusetts, United States; Boston University, Boston, Massachusetts, United States; Tufts Medical Center, Boston, Massachusetts, United States

## Abstract

Mosaic chromosomal alterations (mCAs) are structural alterations that are associated with mortality, age, cancer, cardiovascular disease, and diverse infections. The distribution of mCAs in long-lived subjects and individuals with familial longevity is not well described. We applied MOsaic CHromosomal Alteration (MoChA) caller on genome-wide genotype samples of 2025 centenarians, their siblings, and offspring and 273 unrelated controls from the New England Centenarian Study (NECS) and 3642 subjects with familial longevity and 920 controls from the Long-Life Family Study (LLFS). MoChA utilizes a Hidden Markov Model to detect mCA-induced deviations in allelic balance at heterozygous sites with Log R Ratio and B-allele frequency (BAF) with phased genotype information. We analyzed somatic mCAs in samples with genome-wide BAF phase concordance less than 0.51, LOD score greater than 10, and estimated cell fraction less than 50%. The results in the two studies showed that autosomal mCAs spanning over 100 kbase pairs increase with older age until approximately 102 years. However, the prevalence of the subjects with mCAs tends to plateau after that age, suggesting that the accumulation of mCAs is less prevalent in long-lived subjects. We also found that offspring and siblings of centenarians accumulate less autosomal mCAs (fixed-effect meta-analysis for NECS and LLFS: RR=0.78, p=0.033) compared to unrelated controls. In addition, consistent with results from other studies, mCAs are associated with increased risk for mortality (HR=1.08, p=0.02) and sex (Male RR=1.37, p=4.15e-05), and impact incident events of cancer, dementia, diabetes, and cardiovascular diseases even at extreme old ages.

